# Personal

**Published:** 1899-10

**Authors:** 


### PERSONAL.

Dr. T. B. Carpenter, of Buffalo, announces his removal to 142 North Pearl Street.

Dr. John W. Cameron, has lately joined the ranks of Buffalo physicians. His office is at 159 Woodlawn Avenue. Dr. Cameron is a brother of Dr. J. C. Cameron, professor of obstetrics and diseases of children in McGill University.

Dr. Charles P. Noble announces his removal to 1509 Locust Street, Philadelphia. Consultation hours, daily, 2.30 to 3.30 p. m. Tuesday and Friday, 8 to 11 a. m.

Dr. Carlton R. Jewett, of Buffalo, who has been touring in Europe during a portion of the summer returned September 26th and resumed his professional work.

Dr. Nelson W. Wilson, of Buffalo, has been reappointed acting assistant Surgeon U. S. Army, and assigned to duty at Fort Porter. He has also been designated as examiner of recruits at Buffalo. This appointment is a fitting recognition of Dr. Wilson's services last year in a similar capacity.

Dr. Herbert Mickle, of Buffalo, who has been quite ill during the summer has fully recovered and has resumed his practice.

Dr. Edward J. Meyer, of Buffalo, who recently underwent a severe operation at the General Hospital is reported to be on the road to recovery.

Dr. Herman Mynter sailed for home on September 26th on the Kaiser Wilhelm der Grosse, and is expected in Buffalo on October 4th.

Dr. Alvin A Hubbell, of our associate staff, has just returned from an extended European trip.

